# Impact of the Isolated Cerebral Perfusion Technique for Aortic Arch Aneurysm Repair in Patients with a Shaggy Aorta

**DOI:** 10.3400/avd.oa.21-00128

**Published:** 2022-12-25

**Authors:** Kayo Sugiyama, Hirotaka Watanuki, Masato Tochii, Yasuhiro Futamura, Koki Ishizuka, Katsuhiko Matsuyama

**Affiliations:** 1 Department of Cardiac Surgery, Aichi Medical University Hospital, Nagakute, Aichi, Japan

**Keywords:** shaggy aorta, total arch replacement, isolated cerebral perfusion

## Abstract

**Objective:** Total aortic arch replacement (TAR), particularly in individuals with extensive atherosclerotic alterations, especially shaggy aortas, is more crucial and difficult. The objective of this retrospective investigation was to ascertain if patients with shaggy aortas would respond to modified isolated cerebral perfusion (ICP).

**Materials and Methods:** Between 2015 and 2020, nine individuals with shaggy aortas who received treatment for arch aneurysms were examined. Four and five patients, respectively, who had arch replacement with traditional selective cerebral perfusion (SCP) and modified ICP, were evaluated, and their short- and long-term results were compared.

**Results:** There were no appreciable variations in the postoperative results between patients with traditional SCP and those with modified ICP. Following surgery, one patient developed paraparesis, while two individuals with traditional SCP experienced persistent neurological damage. In patients with modified ICP, there were no postoperative neurological or other problems associated to atherosclerosis; nevertheless, one patient experienced stroke 5 months after surgery.

**Conclusion:** Patients with shaggy aorta may not receive enough brain protection from TAR with standard SCP because single axillary artery perfusion can result in nonphysiological flow and atheroma separation. Even in patients with shaggy aortas, TAR with modified ICP is safe, but late-phase severe adverse cerebrovascular events should be taken into account.

## Introduction

In the past, Amarenco et al. stated that cerebral emboli may be caused by atherosclerotic disease of the aortic arch.^1)^ After cardiovascular surgery, a movable atheroma in the proximal aorta poses a risk for cerebral problems.^2–4)^ The phrase “shaggy aorta,” coined by Hollier et al., refers to the presence of significantly extensive atheromatous disease with diffuse ulcers associated with soft, loosely held debris and a lack of true thrombus.^5)^ A common example of a possible embolic cause of neurologic impairments is a shaggy aorta.^5)^ When there is a shaggy aorta, intraoperative care is crucial in preventing cerebral emboli; the best intraoperative approach, nevertheless, is still debatable.

Shiiya et al. described a novel method for replacing the ascending and transverse aorta by creating selective hypothermic antegrade cerebral perfusion,^6,7)^ while Sawazaki et al. provided an enhanced method for patients with more severe disease.^8)^ For older patients with highly atherosclerotic aortas, modified isolated cerebral perfusion (ICP) during total aortic arch replacement (TAR) is a suitable treatment.^9)^ The purpose of the current retrospective study was to compare the short- and long-term results of traditional selective cerebral perfusion (SCP) and modified ICP in patients with a shaggy aorta in order to ascertain the impact of modified ICP.

## Materials and Methods

Forty-two consecutive patients who had TAR at the Aichi Medical University Hospital between August 2015 and November 2020 were retrospectively reviewed. Nine of 42 individuals with a “shaggy aorta” or severe atheromatous alteration were chosen. The definition of shaggy aorta was based on the concepts introduced by Hollier et al.^5)^ and Amarenco et al.,^1)^ which stipulated that an “atherothrombotic aorta” might be diagnosed when a fragile and spiculated atheroma with a thickness greater than 5 mm was found in the ascending aorta or aortic arch, excluding the aneurysm itself, using computed tomography.

Initially Amarenco et al. promoted the definition of “shaggy” aorta in transesophageal echocardiography^1)^; however, using computed tomography, Hollier et al. established a new characterization of “shaggy” aorta.^5)^ Computed tomography gained popularity within the investigational community since transesophageal echocardiography can be extremely invasive and inconvenient for subsequent study. Four patients underwent standard SCP using perfusion via the ascending aorta and the third part of the right axillary artery between August 2015 and December 2018, while five patients underwent modified ICP using perfusion via the bilateral first part of the axillary arteries and carotid arteries between January 2019 and November 2020. We concentrated on evaluating the patient characteristics, the procedure’s progress, and clinical and neurological results. A basal blood pressure of 149/90 mmHg and continued antihypertensive therapy were considered to be indicators of hypertension. Chronic obstructive pulmonary disease and pulmonary fibrosis requiring particular drugs were considered chronic respiratory diseases. Serum creatinine levels of 177 μmol/L or greater were considered to be indicative of chronic renal disease. Coronary artery disease has been defined as having a history of coronary revascularization.

Early and late clinical results were evaluated. Early results included early death, neurological results, and other atherosclerotic emboli-related problems such as paraplegia, renal failure, intestinal ischemia/necrosis, or peripheral ischemia. The late outcomes included late mortality, major adverse cardiac or cerebrovascular events (MACCEs), and major adverse aortic events (MAAEs). MACCEs were defined as the composite of death, ischemic cardiac events, development of heart failure, or cerebrovascular accidents. Major aortic events or major aortic re-interventions were combined to form MAAEs. Major aortic events included rupture or dissection of the aorta. Major aortic re-interventions included additional thoracic endovascular aortic repair or a major surgical graft revision.

### Definition of neurological deficits

The presence of deficits that persisted at hospital discharge was designated as a permanent neurological deficit based on the definition provided by the Mount Sinai group.^10)^ Delayed awakening, transient loss of orientation, slurred language, poor response to commands, and transient hemiparesis that resolved by hospital discharge were designated as temporary neurological deficits.

### Operative procedures

**Figure 1:**
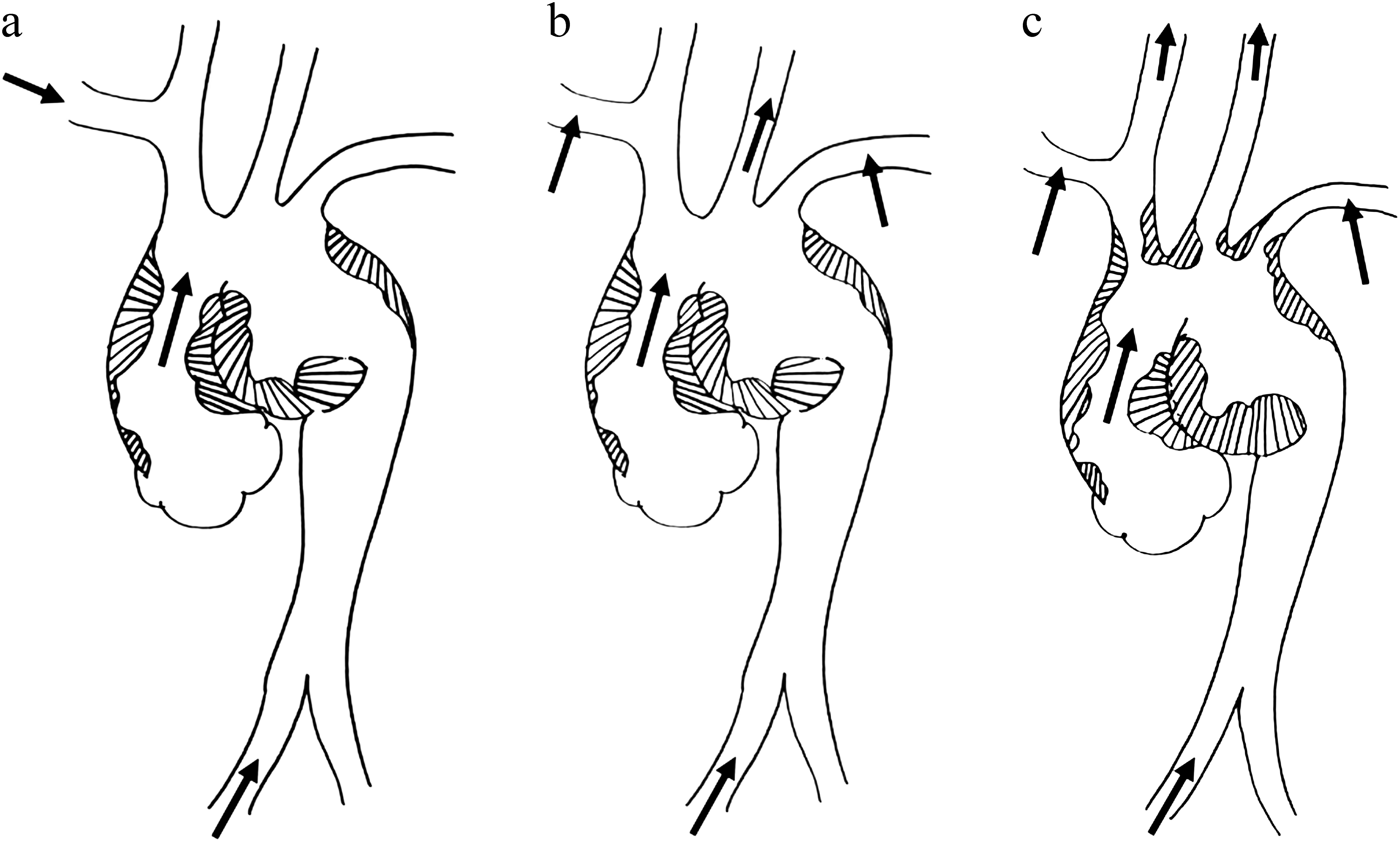
Fig. 1 (**a**) Schematic representation of the standard SCP approach. (**b**) Diagram of the modified ICP technique. (**c**) Schematic depiction of direct carotid artery perfusion at the neck level in the modified ICP technique.

SCP: selective cerebral perfusion; ICP: isolated cerebral perfusion

**Figure 2:**
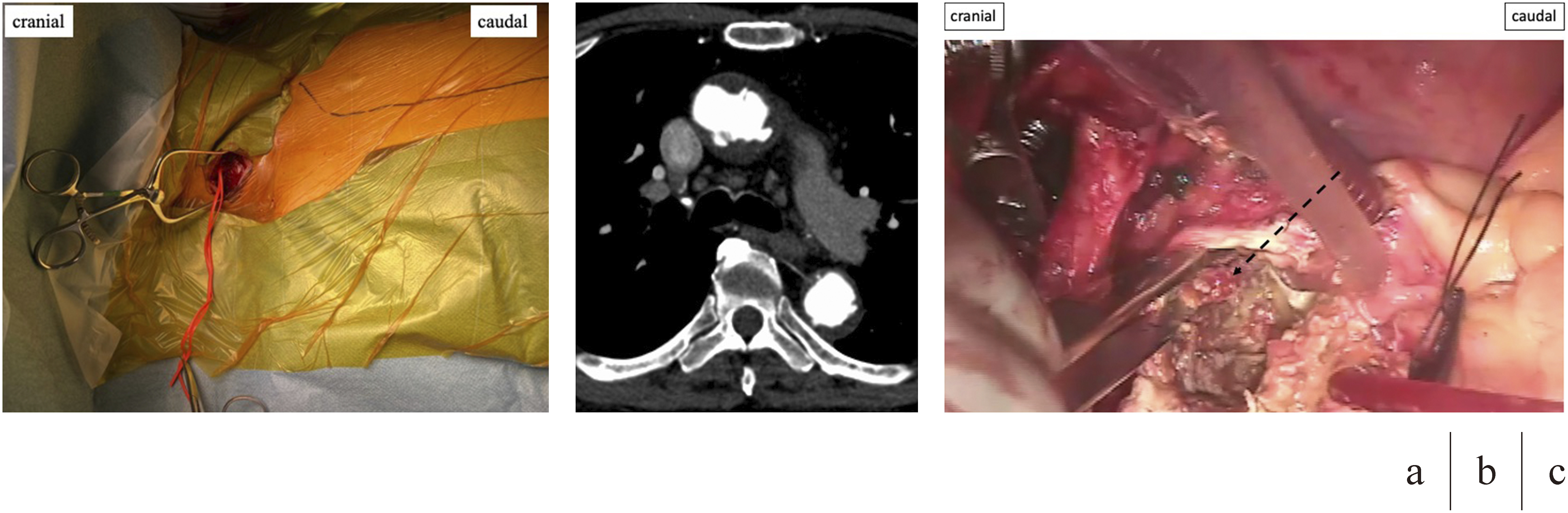
Fig. 2 (**a**) Intraoperative shot demonstrating an incision at the neck level for a second arterial cannula in the right common carotid artery. (**b**) Preoperative contrast-enhanced computed tomography scan of a patient in modified ICP group showing substantial atherosclerotic abnormalities in the ascending aorta. (**c**) Intraoperative photograph demonstrating significant atherosclerotic alterations in the ascending aorta in a patient (**Fig. 1b**) undergoing modified ICP (dotted black arrow).

Surgery for aortic arch was performed through a median sternotomy. Two pumps were used to manage the systemic and cerebral perfusions separately. During conventional SCP, arterial cannulae were placed within the ascending aorta and the third part of the right axillary artery in addition to the femoral artery (**Fig. 1a**). The axillary artery was cannulated directly using a 12-Fr perfusion cannula combined pressure monitoring system with the cut-down technique. One pump was used for systemic perfusion with the ascending aorta and femoral artery, and another pump was used for cerebral perfusion with the right axillary artery at a flow rate of 1.0 L/min. For establishment of the modified ICP procedure, an arterial cannula was added in the first part of the right axillary, left carotid, and left axillary arteries (**Fig. 1b**). An additional arterial cannula was placed in the common carotid artery at the neck level if the orifices of the arch vessels were severely diseased.^11)^ In such cases, the right or left carotid artery was exposed at the beginning of the surgery (**Figs. 1c** and **2a**). The vessel was then cannulated directly using a 12 Fr Perfusion Cannula Combined Pressure Monitoring™ (SUMITOMO BAKELITE, Tokyo, Japan) system with a proximal clamp and cut-down technique. SCP circuit was used to introduce ICP. At this juncture, cerebral perfusion was maintained at a pump flow rate of 1.5 L/min. Cerebral perfusion was started before the commencement of systemic perfusion. Circulatory arrest was introduced when the body temperature reached 25°C. Intra-aortic debris was flushed out using femoral artery perfusion for the ICP and SCP. During circulatory arrest, the SCP flow was 10 mL×kg^−1^×min^−1^. The cerebral perfusion pressure was measured by the tip of each cerebral perfusion cannula, and continuous bilateral cerebral regional oxygen saturation was monitored with INVOS™ Cerebral/Somatic Oximetry Adult Sensors (Medtronic, Minneapolis, MN, USA) throughout the surgery.

ICP: isolated cerebral perfusion

The frozen or fresh elephant trunk technique and simple distal anastomosis was chosen depending on the case. Systemic circulation was resumed after complete establishment of the distal anastomosis. Rewarming was performed after reconstruction of the arch vessels, and anterior SCP was completed. Coronary perfusion was resumed after completion of the proximal anastomosis.

### Statistical procedure

Data are shown as the mean±standard deviation or median (range) for continuous variables, whereas categorical variables are expressed as the number (%) of patients. Categorical variables were analyzed using Fisher’s exact test. Continuous variables were compared using the student’s t-test, while nonparametric variables were analyzed using the Mann–Whitney U test. All data analyses were performed using the JMP 14.1 software (SAS Institute, Cary, NC, USA). Statistical significance was defined as p-values<0.05.

### Definitions

Every procedure was carried out in accordance with the principles of the Helsinki Declaration. The Ethics Committee of Aichi Medical University Hospital approved the study on April 15, 2021 (approval number: 2021-003). All patients provided written informed consent that their clinical data might be used for scientific presentations or publications.

## Results

A total of 42 patients underwent TAR for thoracic aortic arch aneurysms during the study period. Of these, 9 patients (21%) presented with a shaggy aorta, based on the above-mentioned definition. In cases with shaggy aorta, computed tomography demonstrated severe atherosclerotic changes even in the ascending aorta, except for the aneurysm (**Fig. 2b**). The patient characteristics are summarized in **Table 1**. Conventional SCP was used in 4 patients and modified ICP in 5 patients. Two patients in modified ICP required an additional arterial cannula in the common carotid artery because of the severely diseased atherosclerosis. The mean age was 76 years (range, 71–83 years); eight patients were men (89%). All patients had been treated for hypertension, four patients for diabetes mellitus, six for chronic kidney disease, and two for peripheral artery disease. Although seven patients had past smoking habits, no patient had pulmonary emphysema. Five patients had a history of transient cerebrovascular disease, and five had a history of coronary artery disease. The mean age in conventional SCP and modified ICP patients are 74.5±1.89 and 77.6±1.69, which showed no significant difference (p=0.26). There were no significant differences in preoperative characteristics between conventional SCP and modified ICP patients (**Table 1**) except for history of coronary artery disease (p=0.007).

**Table 1:** Table 1 Patient characteristics

	Conventional SCP	Modified ICP	p value
Cases	4	5	
Age	74.5±1.89	77.6±1.69	0.26
Sex (male)	4	4	0.26
HT	4	5	0
DM	1	3	0.29
CKD	2	4	0.34
Smoking	4	3	0.094
COPD	0	0	0
CAD	4	1	0.007
CVD	1	4	0.091
PAD	1	1	0.86

CAD: coronary artery disease; CKD: chronic kidney disease; COPD: chronic obstructive pulmonary disease; CVD: cerebrovascular disease; DM: diabetes mellitus; HT: hypertension; ICP: isolated cerebral perfusion; PAD: peripheral artery disease; SCP: selective cerebral perfusion

**Table 2** provides information on the surgical process and its associated elements (**Fig. 2c**). For the treatment of the distal side of the aorta, five patients were treated with TAR using the frozen elephant trunk technique, two patients using the fresh elephant trunk technique, and one patient was treated using direct anastomosis. One patient with Takayasu’s arteritis was treated with partial arch replacement. Five patients (56%) underwent concomitant coronary artery bypass grafting, and one patient underwent aortic valve repair, tricuspid valve repair, and pulmonary vein isolation. All patients were weaned off cardiopulmonary bypass uneventfully. The operation time and circulatory arrest time were significantly longer in conventional SCP patients (**Table 2**, p=0.020, p=0.029).

**Table 2:** Table 2 Surgical methods

	Conventional SCP	Modified ICP	p value
Cases	4	5	
OPT (minute)	527±31.1	402±27.8	0.020
CPB (minute)	284±18.3	234±16.4	0.080
ACC (minute)	165±12.1	136±10.8	0.12
CA (minute)	66.5±3.84	52.4±3.43	0.029
BT (minute)	24.4±0.18	24.4±0.16	0.97

ACC: aortic cross clamp; BT: bottom temperature; CA: circulatory arrest; CPB: cardiopulmonary bypass; ICP: isolated cerebral perfusion; OPT: operation time; SCP: selective cerebral perfusion

The patients’ postoperative outcomes are listed in **Table 3**. Permanent postoperative neurological deficit due to multiple cerebral infarctions occurred in two patients in the SCP group. However, no significant changes were observed in the cerebral regional oxygen saturation during surgery, even in patients who developed permanent postoperative neurological deficits. Perioperative myocardial infarction occurred in one patient in the SCP group, who also developed paraparesis. Three patients needed transfer to another hospital for rehabilitation after discharge. None of the patients developed bowel necrosis or renal dysfunction. The in-hospital all-cause mortality rate was 0%. The mean follow-up period was 618 (45–1963) days. MACCEs occurred in three patients during the follow-up period. One patient suffered from descending aortic rupture resulting in death, while the remaining two suffered from stroke in the late phase. One patient who underwent conventional SCP did not develop cerebral infarction during the surgery but developed right-sided cerebral infarction 3 months following surgery and another patient who underwent modified ICP developed multiple cerebral infarctions 5 months after surgery. Both patients were treated conservatively without noticeable sequelae. MAAEs occurred in one patient, who developed descending aortic rupture as mentioned above. There were no appreciable variations in the postoperative results between patients with traditional SCP and those with modified ICP (**Table 3**).

**Table 3:** Table 3 Clinical outcomes

	Conventional SCP	Modified ICP	p value
Cases	4	5	
Stroke	2	1	0.34
Spinal	1	0	0.18
ICU stay (day)	2.4±0.64	4.5±0.71	0.064
Hospital day (day)	29.3±4.28	17.0±3.83	0.070
Hospital mortality	0	0	
Delayed mortality	1	0	0.18
MACCE	2	1	0.34
MAAE	1	0	0.18

ICP: isolated cerebral perfusion; ICU: intensive care unit; MAAE: major adverse aortic events; MACCE: major adverse cardiac or cerebrovascular events; SCP: selective cerebral perfusion

## Discussion

In the present study, the number of having coronary artery disease were significantly higher (p=0.007), and the operation time and circulatory arrest time were significantly longer, in conventional SCP patients (p=0.020, p=0.029). However, there were no significant differences in the postoperative outcomes between conventional SCP and modified ICP patients. Additionally, in-hospital death related to thromboembolism were not recorded, however, two patients with conventional SCP developed permanent neurological deficit, and one patient developed paraparesis, postoperatively. Atherosclerosis-related postoperative neurological or other complications were absent in patients with modified ICP; however, one patient developed stroke 5 months postoperatively.

Shaggy aorta, a pathology created by Hollier et al., is a prototypical potential embolic source for neurological deficits.^2,3,5)^ The presence of a mobile plaque in the common carotid artery can lead to embolic events, i.e., atheroembolism, especially when SCP is used for brain protection. Despite the potential advantages of SCP, atherosclerotic stenotic lesions of the extracranial carotid artery can cause neurological deficits.^12)^ Shiiya et al. devised an original ICP technique under deep hypothermic circulatory arrest, as an alternative to SCP, to replace the ascending and transverse aortic aneurysms with severe atherosclerotic changes.^6,7)^ The frequency of cerebral infarction is higher in patients with a shaggy aorta, which necessitated the development of an inverted isolation technique.^7,8)^ In the previous “isolation” technique, SCP was established before systemic perfusion.^7)^ This technique has consequently been changed because technical issues precluded its widespread adoption.^8,9)^ In the modified ICP technique, arterial cannulae are inserted into both axillary arteries at the first part and the left common carotid artery in a non-occlusive manner, and perfusion is initiated through these vessels without the placement of a proximal clamp. Theoretically, aortogenic emboli can never enter the cerebral circulation, and brain circulation is functionally isolated during the modified ICP procedure. We recently introduced a modified ICP technique for cerebral protection during arch repair in patients with a shaggy aorta based on these studies. The current study is the first to report this technique here. In the present study, in-hospital nor late death related to thromboembolism were not recorded, however, two patients with conventional SCP developed permanent neurological deficit, and one patient developed paraparesis, postoperatively. Although there were no significant differences in the postoperative outcomes between conventional SCP and modified ICP patients, the result that perioperative atherosclerosis-related neurological or other complications did not occur in patients with modified ICP is meaningful.

Right axillary artery perfusion is among the promising options for arterial cannulation, the efficacy of which has been reported in previous studies.^13,14)^ However, perfusion is associated with some problems with respect to the hydrodynamic properties. Minakawa et al. performed a streamline analysis of right axillary artery perfusion in the aortic arch aneurysm model.^15)^ The high-velocity flow jet collides the posterior wall of the ascending aorta and enters the ascending aorta with an unexpectedly rapid vortical flow. This type of nonphysiological flow causes detachment of the atheroma, and the debris is consequently washed away into the left common carotid artery. In the present study, permanent neurological deficit and paraparesis occurred in three patients (75%) who underwent conventional SCP. Conventional SCP cannot completely control the dispersal of debris into carotid arteries; modified ICP is an effective method that solves this difficult problem. In addition, direct carotid artery perfusion at the neck level is also suitable for cases with severely diseased orifices of arch vessels.

According to Okada et al., improper cannula choice and a longer cardiac bypass time led to long-term cerebral impairments. They opined that meticulous selection of the cannulation site and cannula type and complete exclusion of the diseased aorta contributed toward the prevention of permanent neurological deficits.^4)^ Similarly, Okita et al. demonstrated that the accurate preoperative and intraoperative imaging of the aortic pathology and complete exclusion of the diseased aorta contributed toward the prevention of permanent neurological deficiency, even in cases with a shaggy aorta.^12)^ As was evidenced by the results of the current study, single axillary artery perfusion may not be reliable and the modified ICP technique may be desirable for brain protection in cases with a shaggy aorta. However, one patient who underwent modified ICP developed stroke 5 months after surgery, while another patient who underwent conventional SCP and did not develop cerebral infarction during surgery also developed stroke 3 months postoperatively. Meticulous follow-up and medical management after surgery are essential, in addition to intraoperative management.

No significant changes were found in the continuous bilateral cerebral regional oxygen saturation in these patients. Monitoring system of cerebral regional oxygen saturation is a reliable modality for detecting changes in cerebral perfusion; however, it may fail to detect fine changes during continuous perfusion with cardiopulmonary bypass.^16)^ Thus, high-risk treatment in patients with a shaggy aorta requires considerable experience and myriad strategies. We endorse the important role of modern specialized aortic centers and experienced aortic surgeons in treating these high-risk patients. Designated specific aortic centers with experienced aortic surgeons can diminish the mortality rate in patients undergoing surgical repair for a shaggy aorta.

Some restrictions applied to this investigation. First, due to the rarity of this illness, the study’s sample size was initially somewhat limited. Second, this study had no randomization and was retrospective, which increases the risk of selection and information bias. Third, during the course of this study, the surgical procedure for aortic arch aneurysms underwent a significant development. For instance, during the course of the current investigation, the way distal anastomoses are treated has changed to include simple anastomosis, fresh and frozen elephant trunk. The reason for the significant difference between both strategies in operation time and circulatory arrest time can be attributed to the above limitation. Further, the catheter interventional technique for atherosclerotic disease has improved, and management protocols and medication treatments have evolved. A multi-institutional study that incorporates propensity matching in its design (for comparison) is needed to resolve these limitations. Further studies are warranted on the underlying brain mechanisms during cerebral perfusion.

Furthermore, as the fourth limitation, this strategy was that distal atherosclerotic emboli to the lower body cannot be prevented, although avoidance of intraoperative stroke can be achieved. A distal embolic protection device is crucial to prevent this complication.

## Conclusion

In patients with a shaggy aorta, TAR with traditional SCP was deemed to be insufficient for brain protection since three of four patients experienced paraparesis or irreversible neurological damage. TAR with modified ICP was performed safely in all five patients with a shaggy aorta without significant embolic problems. However, major adverse cerebrovascular events should be considered in all cases with a shaggy aorta in the late phase.
